# Paediatric Ovarian Dysgerminoma: A Case Report

**DOI:** 10.31729/jnma.7894

**Published:** 2022-11-30

**Authors:** Siddhant Adhikari, Santosh Joti, Parvat Kuwar Chhetri

**Affiliations:** 1 Motherland Hospital, Pepsi-cola, Kathmandu, Nepal; 2 TMSS Medical College, Dhaka-Rangpur Highway, Gokul, Bangladesh

**Keywords:** *case reports*, *dysgerminoma*, *metastasis*, *paediatrics*

## Abstract

Dysgerminoma is the most common malignant germ cell tumour of the ovary. Abdominal pain, abdominal distention, and the presence of a palpable mass are common symptoms at presentation. This is usually detected in youth, before the age of 20 years. Ovarian or adnexal tumours are very rare in patients below the age of 18 years, most of them being functional cysts, only 10% being malignant. Here is a rare case of an 8 years old girl with dysgerminoma who underwent right-sided salpingo-oophorectomy for unilateral involvement with conservation of fertility and now the patient is on chemotherapy as the tumour metastasized to the pre-aortic lymph node.

## INTRODUCTION

The most prevalent malignant ovarian germ cell tumour in females, dysgerminoma accounts for around 2% of all ovarian cancer cases.^1^ Clinical symptoms include abdominal pain, abdominal distention, the existence of a palpable lump, decreased appetite, nausea, and vomiting. The tumour is quite large at the time of diagnosis as in most cases the symptoms are often insidious.^2^ Effective treatment options include conservative surgery, chemotherapy, and postoperative radiation. Surgery to preserve fertility is frequently an option and the overall survival rate is 92.4 per cent.^1,3^ Here, we present a case of an 8-year-old girl with an abdominal mass.

## CASE REPORT

An 8-year-old girl who had not yet reached menarche presented with abdominal distension and a palpable lump in the lower abdomen without associated symptoms. According to her mother, her pregnancy was uneventful and there is no history of carcinoma in her family; there is no history of serious illness or hospital admission and she is a physically healthy-looking child.

During her presentation to our institution, on physical examination there was a mass in the hypogastric region, about 10 x 5 cm^2^ in size, globular in shape, has an irregular surface, ill-defined margin, hard in consistency, moves side to side but not above down and lower limit could not be reached. There was abdominal distension involving the lower part extending up to the umbilical region and percussion note was tympanic all over the abdomen except in the hypogastric region where it was dull. Her haemoglobin level was 11.3 g/dL and her hematocrit (HCT) was 34.3%. Other laboratory reports were unremarkable. An ultrasound scan revealed a soft tissue mass in the lower abdomen measuring 10.7 x 6.1 cm^2^ and the uterus was not marked. The computed tomography (CT) scan revealed a huge soft tissue dense mass lesion measuring about 15.51 x 11.52 x 10.73 cm^3^ in the lower abdomen from the umbilicus to the pelvic cavity with an extension on either side of the midline with an eccentric markedly enhanced solid component measuring about 4.35 x 2.40 cm^2^ at right adnexal region. Considerable mass effects were noted surrounding the organs. Both ovaries could not be identified independently. The post-contrast scan revealed significant enhancement of the larger component. There were no ascites or detectable lymph nodes seen.

Ultrasonography (USG) guided Fine Needle Aspiration Cytology (FNAC) from ovarian mass smears showed a germ cell tumour composed of atypical epithelial cells arranged in small clusters and singly.

Those cells showed enlarged pleomorphic vesicular nuclei, prominent nucleoli, and a moderate amount of clear cytoplasm. The background showed polymorphs, lymphocytes, histiocytes, necrotic debris, and blood suggesting a diagnosis of dysgerminoma. Tumour markers were not available at our institution. All the findings and management options were explained to the patient's mother and she was scheduled for exploratory laparotomy with right-sided salpingo-oophorectomy.

**Figure 1:**
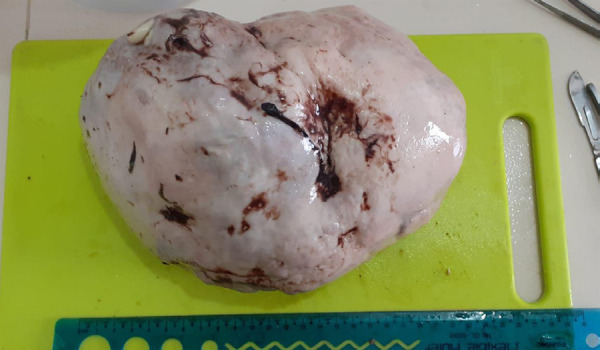
Dysgerminoma.

Per-operative findings showed a huge right, lobulated, cystic ovarian mass whereas the left ovary, fallopian tube, liver, peritoneum and other surrounding structures appeared normal and a tissue sample was sent for histopathology (Figure 1).

**Figure 2:**
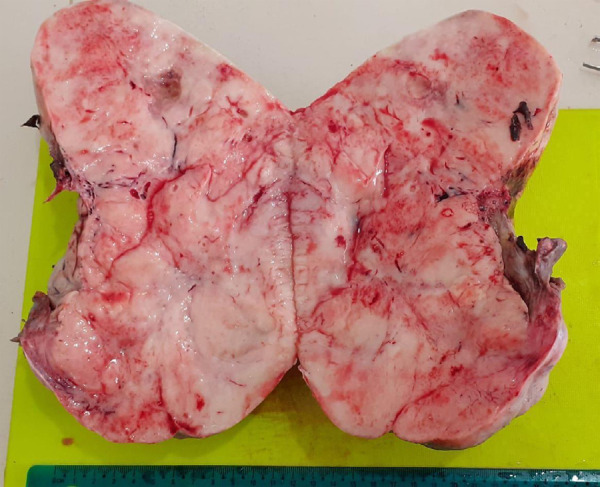
Gross appearance of the cut section of dysgerminoma.

Gross examination revealed a specimen consisting of one nodular piece of tissue measuring 18 x 7 x 6 cm^3^. The cut surface was solid and fleshy (Figure 2).

**Figure 3:**
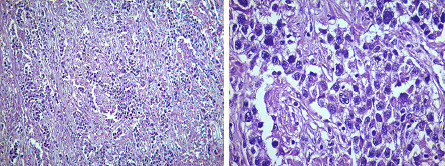
A) Diffuse proliferation of tumour cells arranged in clusters separated by fibrous septa infiltrated with lymphocytes (Hematoxylin and eosin stain, 4X), B) Nest of tumour cells with high nuclear-cytoplasmic (N:C) ratio, vesicular chromatin with prominent nucleoli (Hematoxylin and eosin stain, 40X).

Microscopic examination showed a dysgerminoma arranged in clusters and cords. The cells showed large vesicular cells with well-defined cell borders and prominent nucleoli. Nests of tumour cells separated by fibrous stroma infiltrated with lymphocytes were also seen (Figure 3).

On follow-up after 11 months, an ultrasound scan showed a hypoechoic area about 8 x 5.58 cm^2^ in the upper abdominal cavity pre-aortic location; the peri calyceal system of the right kidney was mildly dilated making an impression of retroperitoneal solid lesion likely of lymph node origin. The findings in USG were a suggestion of metastasis and she is currently on chemotherapy. The patient was administered the first cycles of adjuvant chemotherapy with bleomycin, etoposide and cisplatin (BEP) regimen which she tolerated well. Subsequent cycles will be planned according to the treatment response of the patient which can be evaluated with each cycle of chemotherapy by symptoms, tumour markers, physical exam, and imaging.

## DISCUSSION

A total of 2.6 cases per 100,000 girls are diagnosed with ovarian masses in childhood each year, and 50% of these tumours are malignant. The majority of these tumours are germ cell tumours (85%), followed by epithelial cell carcinoma and sex cord stromal tumors.^4^

In a study where 130 patients were reported as malignant ovarian germ cell tumour, the median age of the patients' were found to be 21 years old, ranging from 4 years to 44 years of age, of which 37.7 % cases were of dysgerminoma,^3^ whereas only 2% of all ovarian cell tumour is dygerminoma.^1^ The patient reported in our case is only 8 years old. Clinical signs of dysgerminoma can range from asymptomatic, abdominal distension, abdomino-pelvic mass, ovarian torsion (adnexal torsion), monthly irregularities, vaginal bleeding, decreased appetite, nausea, vomiting or fever.^2,5^ The tumoral mass often grows quickly and is usually quite large when it is diagnosed.^2^ Similarly, in our case, the patient was asymptomatic and the tumour was quite large at the presentation to us.

Lymphoma is typically the primary differential diagnosis for dysgerminoma. However, lymphoma can be mistaken for dysgerminoma due to its macroscopic and microscopic characteristics. Other differential diagnoses that morphology and immunohistochemistry have ruled out include solid yolk sac tumors, embryonal carcinomas, and Sertoli cell tumors.^1^

Since Human Chorionic Gonadotropin (hCG) tests frequently result in positive results, the diagnosis is frequently mistaken for pregnancy. Pregnancy and dysgerminoma might exist together (20-30%).^6^ As our patient was only 8 years old and non-menstruating rise in hCG would exclude pregnancy. Tumour markers such as lactate dehydrogenase (LDH) may be positive.^6^ Karyotyping is necessary, particularly when a premenstrual girl has a pelvic mass,^6^ but in this case due to poor socio-economic status of the patient and unavailability of tests at our centre, these tests were not carried out.

Although the physical appearance of dysgerminomas varies, in general they are solid, pink to tan to cream-colored lobulated masses, very similar to appearance in this case. Microscopically, there is a monotonous proliferation of large, rounded, polyhedral clear cells that are rich in cytoplasmic glycogen and contain uniform central nuclei with one or a few prominent nucleoli. The tumour cells are histologically identical to seminoma of the testis and closely resemble primordial germ cells of the embryo.^7^ Dysgerminoma is more likely to develop in the right ovary, which was similar in this case. The histogenesis of dysgerminoma may account for its preference for the right ovary, as differentiation of ovarian tissue on the right side occurs more slowly and to a lesser extent than on the left side.^8^

The prognosis and clinical characteristics of the tumour depend on its stage. A five-year survival rate of more than 75% overall is satisfactory (even 90% in stage I), dropping to about 63% in patients with an illness that has spread beyond the ovaries.^9^ Dysgerminoma invades the surrounding tissues directly at the advanced stage. Additionally, it spreads to the level of the pelvic and abdominal peritoneum and distant or local lymph nodes.^10^ Dysgerminoma most frequently metastasizes to the peritoneal cavity, omentum (86%), pelvis, and abdomen, as well as the retroperitoneal lymph node.^7,11^ In addition, extra-abdominal lymph nodes such as supraclavicular, cervical lymph nodes, and as well as other nodes, may be involved.^11-13^ In this case, retroperitoneal solid lesions likely of lymph node origin were seen in USG of the whole abdomen which is suggestive of pre-aortic lymph node metastasis. The patient was asked to be on regular follow-up but she visited to us only 11 months after her surgery which made it difficult to plan for further treatment.

With the availability of modern techniques, USG and CT-guided FNAC can be an ideal modality for the diagnosis of primary and metastatic ovarian neoplasms, as well as the evaluation of recurrent malignant tumours, with significant implications for patient management.^14^ In this patient USG of whole abdomen and USG guided FNAC were done along with CT scan of whole abdomen which suggested the diagnosis of dysgerminoma. In CT and MR imaging, it frequently appears as a large multilobulated purely or predominantly solid mass.^8^

In young patients, the standard treatment for dysgerminoma is usually fertility-sparing surgery with unilateral salpingo-oophorectomy.^6^ Preservation of the contralateral ovary leads to "recurrent" dysgerminoma in 5 to 10% of retained gonads during the next 2 years. Indeed, at least 75% of recurrences develop within the first year of diagnosis.^7^

Due to its rarity, generally being asymptomatic, quite large and extensive during presentation along with high recurrence rate, the treatment is still challenging. Dysgerminomas are inherently radiosensitive; however, pelvic radiotherapy is associated with a high incidence of sterility and with the development of effective chemotherapy regimens, radiotherapy is rarely indicated in children. Bleomycin, Etoposide, and Cisplatin (BEP) is the current treatment recommendation for ovarian dysgerminoma adjuvant chemotherapy.^6^

Dysgerminona is to be considered in prepubertal children presented with abdominal mass. Most cases are asymptomatic and quite large at the time of diagnosis. In CT scan it appears as soft tissue dense mass. Fertility sparing surgery is ideal for young patients with adjuvant chemotherapy for metastasis. Although no signs of metastasis are seen preoperatively and intraoperatively, regular follow up is necessary for early diagnosis and treatment of metastasis cases.
